# Antibiotic treatment induces microbiome dysbiosis and reduction of neuroinflammation following traumatic brain injury in mice

**DOI:** 10.21203/rs.3.rs-4475195/v1

**Published:** 2024-06-11

**Authors:** Hannah Flinn, Austin Marshall, Morgan Holcomb, Leonardo Cruz, Sirena Soriano, Todd J. Treangen, Sonia Villapol

**Affiliations:** Houston Methodist Research Institute; Houston Methodist Research Institute; Houston Methodist Research Institute; Houston Methodist Research Institute; Houston Methodist Research Institute; Rice University; Houston Methodist Research Institute

**Keywords:** microbiome depletion, dysbiosis, brain damage, neuroinflammation, microglia, brain-gut axis

## Abstract

**Background:**

The gut microbiome is linked to brain pathology in cases of traumatic brain injury (TBI), yet the specific bacteria that are implicated are not well characterized. To address this gap, in this study, we induced traumatic brain injury (TBI) in male C57BL/6J mice using the controlled cortical impact (CCI) injury model. After 35 days, we administered a broad-spectrum antibiotics (ABX) cocktail (ampicillin, gentamicin, metronidazole, vancomycin) through oral gavage for 2 days to diminish existing microbiota. Subsequently, we inflicted a second TBI on the mice and analyzed the neuropathological outcomes five days later.

**Results:**

Longitudinal analysis of the microbiome showed significant shifts in the diversity and abundance of bacterial genera during both acute and chronic inflammation. These changes were particularly dramatic following treatment with ABX and after the second TBI. ABX treatment did not affect the production of short-chain fatty acids (SCFA) but did alter intestinal morphology, characterized by reduced villus width and a lower count of goblet cells, suggesting potential negative impacts on intestinal integrity. Nevertheless, diminishing the intestinal microbiome reduced cortical damage, apoptotic cell density, and microglial/macrophage activation in the cortical and thalamic regions of the brain.

**Conclusions:**

Our findings suggest that eliminating colonized gut bacteria via broad-spectrum ABX reduces neuroinflammation and enhances neurological outcomes in TBI despite implications to gut health.

## Background

Traumatic brain injury (TBI) is a prevalent cause of disability and death in adults, frequently resulting from contact sports, military activities, or vehicular accidents [1]. TBI is generally categorized into acute and chronic stages [2], each with unique consequences and inflammatory reactions that influence the secondary inflammatory cascade and the recovery of the patient [3]. Neuroinflammation plays a critical and potentially alterable role in this secondary injury, as supported by animal and human research [4]. Individuals with moderate to severe single TBI might endure a persistent neuroinflammatory state, which can accelerate neurodegenerative processes in the brain. This condition may be exacerbated by a subsequent TBI [5].

The neuroinflammatory response involving microglia and macrophages to brain injury can damage the intestinal mucosa. This damage may alter the gut microbiota’s composition and increase the intestinal barrier’s permeability [6]. Such disruptions can lead to intestinal inflammation, often described as a “leaky gut” [7]. Furthermore, intestinal bacteria can release components, and their byproducts can enter the bloodstream, triggering inflammation through both peripheral and central immune pathways that directly activate the microglia in the brain [8, 9].

Bacteria can influence various neurological processes through the microbiota-gut-brain axis, including myelination, neurogenesis, cytokine release, and microglial activation, including mood and cognition [10, 11]. The diverse microbial populations colonizing the gut have a symbiotic relationship with their host, essential for fermenting undigested carbohydrates, producing short-chain free fatty acids (SCFAs) like acetate, butyrate, and propionate from intestinal bacteria, metabolizing crucial substances, and protecting against pathogens. SCFAs support microglial maturation, innate immune responses [12], and energy metabolism in normal conditions and offer anti-inflammatory benefits [13]. However, disruptions to this symbiosis caused by antibiotic use or illness can lead to dysbiosis.

Gut dysbiosis is often characterized by a reduced abundance or complete loss of SCFA-producing bacteria such as *Faecalibacterium, Christensenellaceae, Collinsella, Roseburia, certain strains of Ruminococcus, Bifidobacterium, Bacteroides, Parabacteroides, Oscillospira, some Clostridium species, and the mucin-degrading bacterium Akkermansia*. There is also often an increase in potential pathogens, including members of the Enterobacteriaceae family, as well as *Campylobacter, Enterococcus, Streptococcus, Staphylococcus, Fusobacterium, Veillonella, Ruminococcus, Megasphaera, and Deltaproteobacteria* [14].

TBI, through the brain-gut-microbiome axis, has been shown to cause changes in microbiota during both the acute and chronic phases [15–20]. Our previous research found that TBI leads to microbiota dysbiosis, with a notable decrease in *Lactobacillus gasseri, Ruminococcus Flavefaciens*, and *Eubacterium ventriosum* [21]. Besides, the *Firmicutes-to-Bacteroidetes* ratio decreased as part of the stress response after TBI [22]. Clinical findings from a recent study suggest that within 72 hours after severe trauma, patients with multiple injuries showed a decline in beneficial bacteria but an increase in *Clostridiales* and *Enterococcus* [23].

Antibiotics (ABX) are frequently administered in intensive care units to prevent infections and sepsis in TBI patients, thereby reducing injury-related and hospital-acquired infections [24]. Early administration of ABX is associated with improved survival in TBI patients [25], and improves the TBI outcome [26]. In preclinical studies, a standard approach to deplete enteric bacteria with minimal systemic absorption is to administer a broad-spectrum ABX cocktail [27]. ABX can have rapid and potentially lasting effects on the microbiota. However, ABX can lead to gut microbiota dysbiosis, associated with altered signaling molecules and cognitive impairments [28]. The administration of ABX in TBI patients is not without some risk due to potential reductions in healthy gut microbiota and its link to increased ABX resistance among bacteria [29]. Further research is needed to understand the full impact of ABX on recovery from brain injuries and its relationship with microbiome dysbiosis.

In this study, we administered a broad-spectrum antibiotic cocktail to young male mice and noted that the elimination of intestinal bacteria leads to dysbiosis, decreased neuroinflammation, and enhanced outcomes after TBI. These results underscore the importance of further exploring the connections between the gut and brain microbiomes to identify possible therapeutic strategies for TBI patients.

## Methods

### Mice, administration of antibiotics and TBI model.

**Figure 1:**
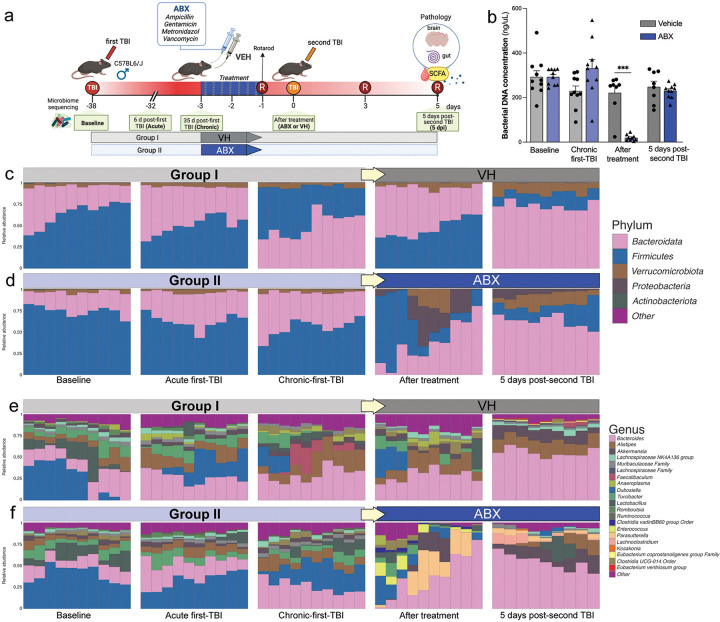
Effects of antibiotic treatment on the microbiome composition in a model of traumatic brain injury (TBI). **(a)** Schematic overview of the experimental design. Male C57BL/6J mice (n=10/group) were subjected to a controlled cortical impact (CCI) injury to induce a first TBI. Microbiome sequencing was conducted at baseline, 6 days post-first TBI (acute phase), 35 days post-first TBI (chronic phase), after a course of antibiotics (ABX) or vehicle (VH) treatment via oral gavage, and 5 days post-second TBI. The ABX cocktail consisted of ampicillin, gentamicin, metronidazole, and vancomycin. Rotarod performance was assessed as a measure of motor coordination and balance, and SCFA were measured in serum at 5 days post-TBI (dpi). **(b)** Quantification of bacterial DNA concentration in fecal samples at different time points, comparing the VH and ABX-treated groups. Data are represented as mean ± SEM; ***p<0.001. **(c-f)** Relative abundance of bacterial taxa at the phylum and genus levels for two distinct experimental groups (I and II). Each panel represents a different time point: Baseline, Acute first-TBI, Chronic first-TBI, After treatment (VH), and 5 days post-second TBI. Group I received VH treatment, while Group II received ABX. **(c, d)** Relative abundance at the phylum level, color-coded for major phyla including *Bacteroidota, Firmicutes, Verrucomicrobiota, Proteobacteria, Actinobacteria,* and others. **(e, f)** Relative abundance at the genus level, showing detailed shifts in microbial composition with a diverse set of genera color-coded for ease of differentiation. Both sets of bar charts reveal the impact of ABX on microbial diversity and the subsequent changes following a second TBI (5 dpi).

Young adult (14-week-old) C57BL/6J male mice (Jackson Laboratories, Bar Harbor, ME) were housed at the Houston Methodist Research Institute animal facilities under a standard 12-hour light and dark cycle with access to food and water ad libitum. All *in vivo* experiments were approved by the Institutional Animal Care and Use Committee (IACUC) at Houston Methodist Research Institute, Houston (Texas, USA). To achieve microbiome depletion, an antibiotic (ABX) cocktail was made consisting of ampicillin (1 mg/mL), gentamicin (1 mg/mL), metronidazole (1 mg/mL), and vancomycin (0.5 mg/mL), dissolved in autoclaved drinking water. The cocktail was administered to ABX-treated male mice via oral gavage in a dosage of 200 mL for 2 consecutive days. The vehicle (VH)-treated mice underwent all procedures except microbiota depletion and were administered water as control VH via oral gavage. Experimental groups consisted of an ABX and VH treatment (n = 10 per group). Mice from both treatment groups were anesthetized with isoflurane before receiving an initial controlled cortical impact (CCI) injury on the left hemisphere at the primary motor and somatosensory cortex using an electromagnetic Impact One stereotaxic impactor (Leica Microsystems, Buffalo Grove, IL, USA). The impact site was localized at 2 mm lateral and 2 mm posterior to Bregma with a 3 mm diameter flat impact tip. The impact was made at a velocity of 3.2 m/s and impact depth of 1.5 mm. As specified in our earlier studies, these parameters were determined to induce an inflammatory response post-TBI [2, 30]. A second CCI was performed following the same parameters 38 days after the initial injury. Mice were anesthetized and sacrificed 5 days post-injury (dpi) (after the second CCI), and brains, blood, and small intestines were collected for further analysis (Fig. 1a).

#### Fecal Microbiome DNA extraction for 16S rRNA Sequencing

Gut microbiome DNA concentrations were obtained by collecting stool samples from the VH and ABX groups (Fig. 1a). After collection, stool samples were stored at −80°C. Genomic bacterial DNA was extracted from frozen stool samples using the QIAamp PowerFecal Pro DNA Kit (Qiagen, Germantown, MD). Bead beating was implemented in three cycles for DNA extraction, each lasting one minute at a speed of 6.5 m/s and a rest period of five minutes between cycles. This mechanical disruption was conducted using a FastPrep-24 system (MP Biomedicals, Irvine, CA). Following the bead-beating process, DNA isolation was completed according to the manufacturer’s instructions. The concentration of the extracted genomic DNA was then measured using a DS-11 Series Spectrophotometer/Fluorometer (DeNovix, Wilmington, DE). Extracted genomic bacterial DNA from collected fecal samples was analyzed for microbiota colonization and diversity by 16S rRNA gene compositional analysis. Illumina MiSeq was performed using the adapters and single-index barcodes so that the polymerase chain reaction (PCR) products could be pooled and sequenced directly, targeting at least 10,000 reads per sample [33]. Primers used for the 16S V1-V3 amplification were 27F (AGAGTTTGATYMTGGCTCAG, where Y = C (90%) or T (10%); M = A (30%) or C (70%) and 534R (ATTACCGCGGCKGCTGG, where K = G (10%) or T (90%) [34]. Amplicons were generated using primers corresponding to the V1-V3 variable regions, and the PCR products were purified. Subsequently, sequencing libraries for the V1-V3 target were constructed following the instructions provided by the Illumina MiSeq system with end products of 300 bp paired-end libraries.

#### Microbiome Data Analysis

Raw data files in binary base call (BCL) format were converted into FASTQs and demultiplexed based on the single-index barcodes using the Illumina ‘bcl2fastq’ software. Demultiplexed read pairs underwent quality filtering using bbduk.sh (BBMap version 38.82), removing Illumina adapters, PhiX reads, and sequences with a Phred quality score below 15 and length below 100 bp after trimming. 16S V1-V3 quality-controlled reads were then merged using bbmerge.sh (BBMap version 38.82), with merge parameters optimized for the 16S V1-V3 amplicon type (vstrict = t qtrim = t trimq = 15). Further processing was performed using nf-core/ampliseq version 2.8.0 of the nf-core community workflows, implementing reproducible software environments from the Bioconda and Biocontainers projects [35–38]. Data quality was evaluated with FastQC (version 0.12.1) and summarized with MultiQC (version 1.18) [39]. Sequences were processed sample-wise (independent) with DADA2 (version 1.28) to eliminate any residual PhiX contamination, trim reads (forward reads at 275 bp and reverse reads at 265 bp; reads shorter than this were discarded), discard reads with > 2 expected errors, to correct errors, to merge read pairs, and to remove PCR chimeras. After clustering, 2097 amplicon sequencing variants (ASVs) were obtained across all samples. Between 6.77% and 34.22% of reads per sample (average 15.3%) were retained. The ASV count table contained a total of 1663253 counts, at least 4360 and at most 36800 per sample (average 16972). Taxonomic classification was performed by DADA2 and the database ‘Silva 138.1 prokaryotic SSU’ [41]. ASV sequences, abundance, and DADA2 taxonomic assignments were loaded into QIIME2 (version 2023.7) [42]. Within QIIME2, the final microbial community data were collected into an .rds file found within the phyloseq (version 1.44) folder of the nf-core ampliseq output [43]. The resulting phyloseq data frame object was merged with the phylogenetic tree calculated within QIIME2. The phyloseq object was used in the creation of alpha and beta diversity plots, PERMANOVA calculations with vegan::adonis2 (version 2.6–5), relative abundance bar plots with microViz (version 0.12.1), nextflow, and R (version 4.3.3) scripts, and differentially abundant taxa calculations using ANCOMBC2 [44]. OTU-based analysis was also performed using Lotus2 [31]. Samples were quality filtered, demultiplexed, and prepared for downstream processing using sdm. Taxonomic alignment to the ‘Silva 138.1 prokaryotic SSU’ was performed using the RDPclassifer [32]. OTUs were clustered using VSEARCH and a final phylogenetic tree was constructed from a MAFFT multiple sequence alignment using FastTree2 [33–35]. Resulting OTU data analysis and visualization was performed using the. Rdata file produced from the Lotus2 workflow with the same R code written for the ASV analysis (Supplementary Fig. 1). All scripts used in both data processing and visualization can be found at https://github.com/microbemarsh/abx_depletion. Data are stored in the SRA database from NIH with the BioProject number: PRJNA1104663.

### Serum SCFA analysis.

SCFAs were analyzed at 5 dpi by derivatization procedure. 40 μL of serum was added to 40 μL of acetonitrile, vortexed, and centrifuged. 40 μL of the supernatant, 20 μL of 200 mM 12C6- 3-Nitrophenylhydrazine (3NPH), and 120 mM 1-Ethyl-3-(3-dimethylaminopropyl)carbodiimide (EDC) were combined. 20 μL of hydrochloric acid was added and incubated for 30 min at 40°C. The resulting mixture was cooled and made up to 1.91 mL with 10% aqueous acetonitrile. 5 μL of the sample was injected into liquid chromatography-tandem mass spectrometry (LC-MS/MS). SCFAs were separated using mobile phases 0.1% formic acid in water (mobile phase A) and 0.1% formic acid in acetonitrile (mobile phase B). Separation of metabolites was performed on Acquity UPLC HSS T3 1.8 um (2.1×100mM). The SCFA were measured in ESI negative mode using a 6495 triple quadrupole mass spectrometer (Agilent Technologies, Santa Clara, CA) coupled to an HPLC system (Agilent Technologies, Santa Clara, CA) with multiple reaction monitoring (MRM). The acquired data was analyzed using Agilent Mass Hunter quantitative software (Agilent Technologies, Santa Clara, CA). Raw peak intensity for each SCFA was normalized by sum, log transformed, and auto-scaled (mean centered and divided by standard deviation).

#### Rotarod Test

The sensorimotor function was evaluated by means of a Rotarod behavior test using the system from Ugo Basile (Gemonio, Italy). All mice underwent an initial training session of 3 trial runs, two days before behavior testing began. Following the completion of training, mice were assessed at the following time points: 24 h after ABX/VH treatment, 3 dpi, and 5 dpi to assess motor function following the second TBI. Mice were placed on the stationary rod and allowed to explore for 30 sec. The stationary rod was then rotated, accelerating from 4 to 40 rpm for 5 min. Each trial ended when the animal fell off the rod. The latency to fall was recorded, and the mean value was established for each mouse. All behavior testing was conducted by an experimenter blinded to the animal groups.

#### Cresyl Violet staining

Fixed whole brain samples were sectioned using a cryostat (Epredia Cryostar NX50, Fisher Scientific, Waltham, MA, US), in which 15 μm slices were processed and mounted directly onto glass slides or submerged in a free-floating cryo-protective solution (30% sucrose, 1% polyvinylpyrrolidone, 30% ethylene glycol, and 0.01M PBS). For immunohistochemical usage, collected sections were taken at coronal planes from the frontal cortex throughout the dorsal hippocampus. A cresyl-violet solution was prepared under a ventilated hood by combining 0.1% cresyl-violet (Sigma-Aldrich, St. Louis, MO, US), acetic acid, and distilled water. Pre-mounted brain sections were stained with cresyl violet solution for 10–20 min before dehydrating in ethanol dilutes. Lastly, slides were submerged in xylene before being covered with a xylene based Permount (ThermoFisher Scientific) mounting media and coverslipped. The lesion volume was calculated as a percentage of the lesion area. Collected data was then averaged for each of the 9–12 brain sections per slide using ImageJ software.

### Immunohistochemistry, cell death assay and quantitative analysis.

Free-floating brain sections were first washed with a series of PBS and 0.5% PBS-Triton X-100 (PBS-T) before applying a 3% normal goat serum (NGS, #1000, Vector Laboratories, Burlingame, CA) blocking solution for 1 hour at room temperature (RT). A primary antibody solution made from blocking solution (PBS-T and 3% NGS) and dilutions of the following primary antibodies: anti-rabbit Iba-1 (1:500, Wako), anti-mouse CD68 (1:200, Biorad), anti-rat F4/80 (1:200, R&D Systems), anti-rabbit P2Y12 (1:500, Anaspec), anti-rabbit GFAP (1:500, Dako), and anti-rat Ly6B2 (1:500, Biorad), were incubated at 4°C overnight. The next day, brain sections were washed in PBS-T 3×5min and incubated with the corresponding anti-rabbit or anti-mouse Alexa Fluor 568-conjugated and anti-rat Alexa Fluor 488-conjugated IgG secondary antibody (all 1:1000, Thermo Fisher Scientific, Waltham, MA, USA) for 2 hours at RT. Samples were washed with distilled water 3×5min before being counterstained with DAPI solution diluted in PBS (1:50,000, Sigma-Aldrich) for 5 min. Sections were mounted and cover slipped using Tris Buffer mounting medium (Electron Microscopy Sections, Hatfield, PA). Cell death was evaluated separately using the Fluorescence *In Situ* Cell Death Detection kit (Roche Diagnostic, Indianapolis, IN, US). Brain sections were analyzed for DNA strand breakage using Terminal deoxynucleotidyl transferase dUTP nick end labeling (TUNEL) following the manufacturer’s instructions. All histological images were acquired on a Nikon fluorescence microscope (Eclipse Ni-U, Melville, NY, USA) and confocal imaging system (Leica Microsystems, Deerfield, IL, USA). Areas of interest were the somatosensory cortex and thalamus regions of the brain. For the quantitative analysis of immunolabeled sections, we employed unbiased, standardized sampling methods to evaluate tissue areas in the cortex and thalamus that exhibited positive immunoreactivity. To measure the number of Iba-1 positive cells, we analyzed an average of five single-plane sections from the lesion center (ranging from − 1.34 to −2.30 mm from bregma) for each animal, blind to the conditions, across each brain region. Within each region, all cells positive for Iba-1 CD68, F4/80, P2Y12, and Ly6B2 in five specific fields in the cortex and two specific fields in the thalamus (x20, 151.894 mm2) near the impact site were counted. For proportional area measurements, we quantified the extent of the reaction for microglial and astroglial cells as the percentage of the area occupied by immunohistochemically stained cellular profiles within the injured cortex and thalamus regions. The data were presented as the percentage of area showing Iba-1 or GFAP positive immunoreactivity relative to the total studied area. We performed a quantitative evaluation of immunoreactive regions for Iba-1 and GFAP across 15 cortical and thalamus areas at the impact level. Quantitative image analysis of the staining’s in the cortical and thalamic regions was performed using ImageJ64 software (NIH, Bethesda, MD, USA) as previously described [30] for inversion, thresholding, and densitometric analysis. The threshold function was utilized to set a black and white threshold corresponding to the imaged field, subtracting the averaged background. The “Analyze Particles” function was then used to calculate both the total area of positive staining and the proportion of the total area.

### Microglia Morphological analysis.

Immuno-stained anti-rabbit Iba-1 brain sections were imaged at a 40x oil objective using a confocal imaging system (Leica Microsystems, Deerfield, IL, USA) to further investigate microglia structure and morphology. Microglia in the somatosensory cortex and thalamus were reconstructed with Neurolucida morphometric software (MBF Biosciences, VT, USA) by using a 3D sectional plane. First, microglia somas were individually labeled to identify central points of each microglia. Dendrite branch mapping was then performed using the program’s tracing tool. Once mapped, tracings were rendered into a 2D diagram using NeuroExplorer software (MBP Biosciences, VT, USA). A Sholl analysis was performed to assess cell complexity in relation to soma size and distance. Concentric circles spaced 5 μm apart, originating from the soma, were placed over each microglia. The number of dendrite branches that intersected the radius, the average dendrite length, the number of nodes (branching points), and the average surface area of individual microglia were measured as a function of the distance from the cell soma for each radius (Fig. 5c–f). The total data was graphed using GraphPad Prism 8 software (GraphPad; San Diego, CA, US) and mapped for significance in the area under the curve (AUD).

#### Gut Histology Staining

The small intestines of ABX and VH grouped mice were isolated and fixed in 4% paraformaldehyde for 48 hours before being transferred to a 70% ethanol solution for dehydration. Samples were processed using a Shandon Exelsion ES Tissue Processor and embedded in paraffin on a Shandon HistoCenter Embedding System, as specified by the manufacturer’s standard processing and protocols. Samples were sectioned at 5 μm thickness and mounted onto glass slides. Intestinal samples were deparaffinized in xylene before being rehydrated in water and then stained with a hematoxylin solution for 6 hours at 60–70 °C. The tissue was then rinsed with tap water to remove excess stain before being treated with 0.3% acid alcohol in water. It was then used to differentiate the tissue before being rinsed in tap water and stained with eosin for 2 min. Slides were then rinsed and mounted with a xylene-based Permount mounting medium and allowed to dry overnight. Alcian Blue staining was then performed to evaluate mucin production facilitated by goblet cells in the intestines. Intestinal samples were deparaffinized in xylene before being dehydrated in dilutions of ethanol and water. Slides were washed in distilled water before applying the Alcian blue solution for 30 minutes. Excess stain was removed using tap water before Nuclear Fast Red Solution was applied for 5 min. Samples were then rinsed and dehydrated before being dipped in xylene and mounted as previously described.

#### Statistical analysis

Statistical significance from SCFAs analysis was determined using multiple comparison t-tests within MetaboAnalyst6 [57]. Orthogonal Partial least squares discrimination analysis (oPLS-DA) calculation and visualization was performed using the MetaboAnalystR [56, 58] package within R (version 4.3.3). Correlation heatmaps of key bacterial taxa to SCFAs were performed using normalized metabolomic data and microbial counts within a custom R script at https://github.com/microbemarsh/abx_depletion.

Analysis of the rotarod test and histochemical/immunofluorescence utilized a one-way ANOVA followed by a Tukey’s multiple comparison to compare the time after injury and sex as the independent variables. A post hoc test with Bonferroni multiple test correction was then applied. All data in the study were presented as mean ± SEM. *p < 0.05, **p < 0.01, ***p < 0.001, and ****p < 0.0001 were considered statistically significant. GraphPad Prism 8 Software (GraphPad; San Diego, CA, US) was used for statistical analysis.

## Results

### ABX treatment induce shifts in gut taxonomy composition.

The experimental design involved inducing a first TBI, followed by microbiome sequencing at various timepoints: baseline, 6 days post-first TBI (acute phase), 35 days post-first TBI (chronic phase), after a course of ABX or VH, and 5 days post-second TBI (Fig. 1a). Bacterial DNA concentration in fecal samples showed significant decreases following ABX treatment, exhibiting lower DNA concentrations than VH after treatment (Fig. 1b, ***p < 0.001) ensuring the effectiveness of the ABX depletion.

The relative abundance of bacterial taxa at the phylum and genus levels are represented in two experimental groups: Group I (VH) had relatively stable microbiomes across the time points, while Group II (ABX) showed significant shifts in composition (Fig. 1c–f). TBI induced the change of gut microbiota components in both groups. Relative abundance at the phylum level indicated changes in the balance of major phyla like *Bacteroidota, Firmicutes, Verrucomicrobiota, Proteobacteria, and Actinobacteria* (Fig. 1c). Relative abundance at the genus level (Fig. 1e, f) demonstrated significant changes following ABX treatment, revealing a more diverse microbial composition, with notable alterations post-second TBI.

### Gut bacterium diversity and concentrations shift following TBI and ABX treatment.

**Figure 2:**
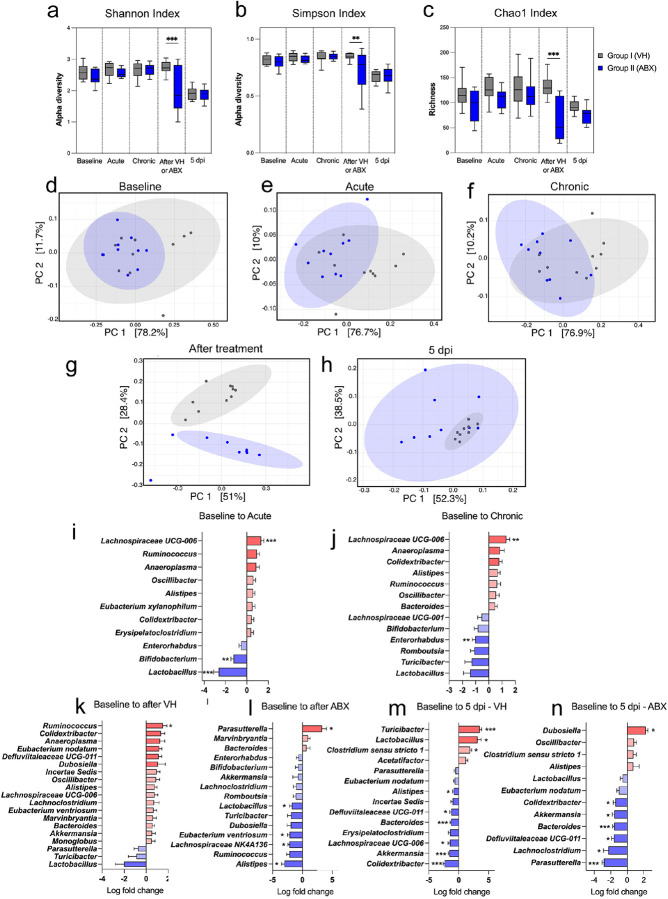
Changes in Microbiome Diversity and Genus level Abundance Changes after ABX Treatment following Traumatic Brain Injury (TBI) in mice. **(a-c)** Boxplots illustrating alpha-diversity indices for two groups: Group 1 (VH-treated) and Group 2 (ABX-treated), at various time points—Baseline, Acute TBI, Chronic TBI, After VH/ABX treatment, and 5 days post-second TBI (5 dpi). **(a)** Shannon, **(b)** Simpson, and **(c)** Chao1 demonstrate significant changes in diversity, with Group 2 showing decreased diversity post-ABX treatment within all indices. (*p<0.05, ** p<0.01, ***p<0.001, n=10/group). **(d-h)** Principal coordinate analysis (PCoA) plots show clustering of microbial communities at different time points. Each plot corresponds to Baseline **(d)**, Acute post-first TBI **(e)**, Chronic post-first TBI(f), After VH/ABX treatment **(g)**, and 5 dpi **(h)**, reflecting shifts in microbial community composition. **(i-l)** ANCOMBC2 log-fold change in abundances from Baseline to different time points: **(i)**Baseline to Acute, **(j)** Baseline to Chronic, **(k)** Baseline to After ABX **(l)** Baseline to 5 dpi **(m)** Baseline to 5 dpi for VH group and**(n)** Baseline to 5 dpi for antibiotic group. Each plot lists genera with significant changes in abundance, with red indicating an increase and blue a decrease in abundance relative to Baseline. (*q<0.05, ** q<0.01, ***q<0.001, n=9/group).

Microbiome diversity and genus abundance changes were analyzed. Alpha-diversity was assessed using three statistical indices; Shannon (Fig. 2a), Simpson (Fig. 2b), and Chao1 (Fig. 2c), to measure the observed bacterial richness. The results indicated decreased alpha diversity in the ABX group compared to VH, with significant reductions in the Shannon and Chao1 indices after ABX treatment (*p < 0.05, **p < 0.01, and ***p < 0.001, respectively). These changes were evident across time points, from baseline to 5 dpi. The beta diversity was measured by plotting the weighted UniFrac distance using principal coordinate analysis (PCoA) to quantify the variability among bacteria communities (Fig. 2d–h). It revealed a trend of separation by treatment. Each plot represents a specific stage, showing noticeable differentiation between VH and ABX groups. Specifically, plots for baseline (Fig. 2d), acute (Fig. 2e), chronic (Fig. 2f), after VH/ABX treatment (Fig. 2g), and 5 dpi (Fig. 2h) highlight these changes in microbial diversity. The red bars indicate increased abundance, while the blue bars signify decreases relative to baseline. Furthermore, we found taxa with significantly different abundance levels among the groups. This suggested that variations were detectable across all taxonomic ranks, from phylum to genus, and distributed across all phyla. Acute TBI induced the change of gut microbiota components, including a decrease of *Lactobacillus* (LFC = −2.63, q = 1.51e-4) and *Bifidobacterium* (LFC = −1.25, q = 1.18e-3) coinciding with a significant increase in *Lachnospiraceae* UCG-006 (LFC = 1.33, q = 6.31e-4). The chronic inflammatory stage resulted in less differential bacteria when compared to the baseline than during the acute stage, with *Lachnospiraceae* UCG-006 (LFC = 1.33, q = 6.31e-4) increasing again and significant reductions seen within *Enterorhabdus* (LFC = −1.03, q = 2.76e-3) and *Romboutsia* (LFC = −1.09, q = 0.04). The fecal microbiomes of mice after ABX treatment were significantly decreased in both counts and diversity when compared to their VH cohort, which still saw increased diversity rates as seen by the number of taxa increasing in abundance with *Ruminococcus* (LFC = 1.51, q = 0.03) significantly increasing in the VH group however the only genus to significantly increase in abundance after the ABX administration was *Parasutterella* (LFC = 3.28, q = 0.01). Genera that were reduced considerably after the ABX treatment were *Alistipes* (LFC = −3.01, q = 1.74e-4), *Lachnospiraceae NK4A136* (LFC = −2.14, q = 3.3e-3), *Eubacterium ventriosum* (LFC = −2.11, q = 3.50e-3), and *Lactobacillus* (LFC = −1.75, q = 0.02). Lastly, after a second TBI, both mouse cohorts experienced great change at the 5 dpi timepoint. Figure 2m shows the impact of the second TBI on the VH group with significant increases in *Turicibacter* (LFD = 3.52, q = 9.50e-6), *Lactobacillus* (LFC = 3.14, q = 5.33e-3), and *Clostridium sensu stricto 1* (LFC = 1.88, q = 1.14e-3) meanwhile significant decreases in *Colidextribacter* (LFC = −2.35, q = 3.57e-5), *Akkermansia* (LFC = −1.54, q = 3.40e-5), *Lachnospiraceae* UCG-006 (LFC = −1.46, q = 0.02), *Bacteroides* (LFC = −1.27, q = 1.04e-5), *Defluviitaleaceae* UCG-011 (LFC = −1.23, q = 3.3e-3), and *Alistipes* (LFC = −0.83, q = 6.40e-3). Within the ABX-treated cohort, a large number of taxa were also seen to be reduced when compared to the baseline however the taxa were slightly different with the only significant increase being *Dubosiella* (LFC = 2.22, q = 1.01e-3) and significant decreases in *Parasutterella* (LFC = −2.83, q = 1.10e-7), *Lachnoclostridium* (LFC = −2.29, q = 0.03), *Defluviitaleaceae* UCG-011 (LFC = −1.61, q = 1.83e-3), *Bacteroides* (LFC = −1.56, q = 2.62e-5), Akkermansia (LFC = −1.54, q = 1.10e-3), and *Colidextribacter* (LFC = −1.44, q = 8.03e-3). These results suggest that antibiotic treatment following TBI significantly impacts microbial diversity and genus abundance, leading to notable shifts in microbial composition that could have implications for brain-gut interactions and post-TBI recovery.

### ABX treatment did not alter the SCFA profiles after TBI.

**Figure 3:**
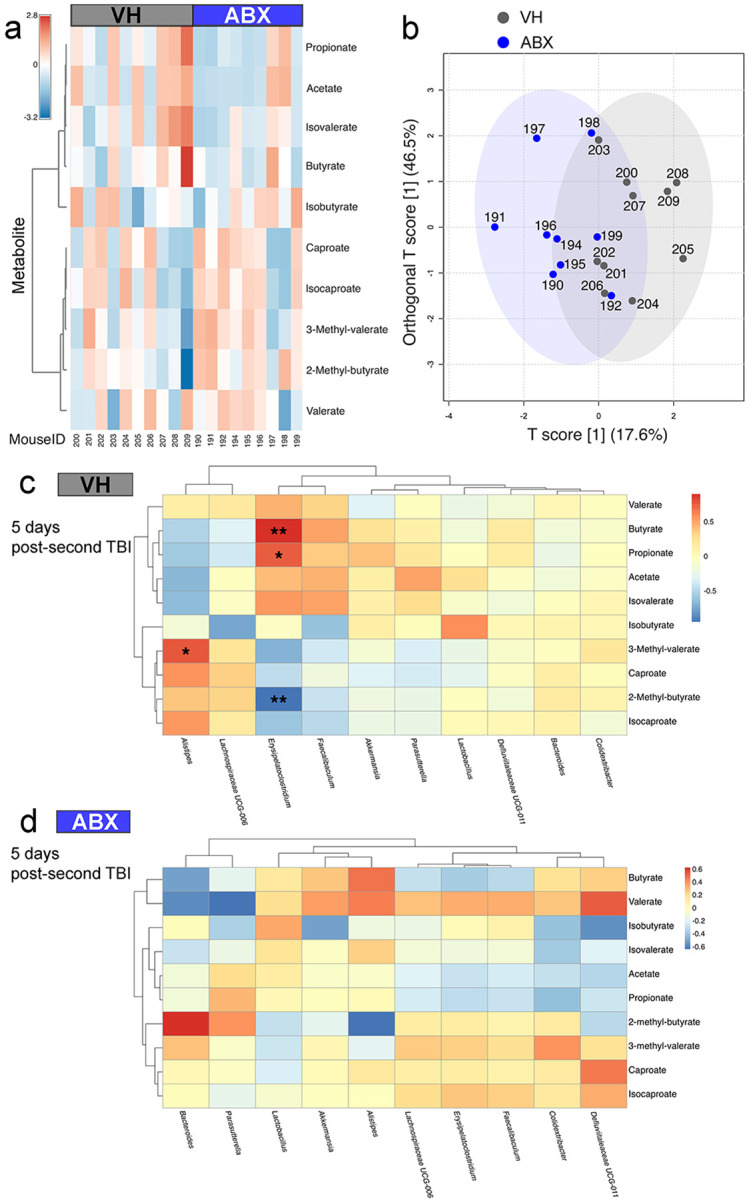
Short-Chain Fatty Acid (SCFA) Profiles and Bacterial Genus Correlations are maintained in TBI mice after ABX treatment. **(a)** Heatmap displaying normalized peak intensity of various SCFAs in blood samples from two groups of mice, vehicle (VH) and antibiotics (ABX) treated. Each row represents a different SCFA, while each column corresponds to an individual mouse, identified by unique Mouse IDs. **(b)** Orthogonal partial least squares differential analysis (oPLS-DA) of SCFAs between Group I and Group II mice. Each point represents an individual mouse sample, with each axis reflecting maximal variance in SCFA levels between the groups. The percentage of variance explained by each component is indicated in parentheses. Pearson’s correlation heatmap coupled with hierarchical clustering shows the relationship between key bacterial genera and SCFA production for Group I **(c)** and Group II mice **(d)**. Columns represent bacterial genera and rows represent SCFAs, asterisks indicate significant correlations controlling for false discoveries using the Benjamini-Hochberg procedure. (*q<0.05, ** q<0.01, ***q<0.001, n=9/group).

SCFA profiles and bacterial genus correlations were examined following ABX treatment and VH in a TBI mouse model. The heatmap shows the normalized peak intensity of various SCFAs in blood samples from two groups of mice, VH and ABX-treated (Fig. 3a). Each row corresponds to a specific SCFA, and each column represents an individual mouse identified by unique Mouse IDs. Differences in SCFA profiles are evident, with each group displaying a distinct pattern of SCFA production. Orthogonal partial least squares differential analysis (oPLS-DA) of SCFAs (Fig. 3b) between VH and ABX mice showed clear separation, with each point representing an individual mouse sample. The axes represent the maximal variance in SCFA levels between groups, indicating significant differences in SCFA profiles. The percentage of variance for the T score is 17.6%, and for the orthogonal T score is 46.5%, providing insights into the underlying variance structure. Additionally, Pearson’s correlation heatmap and hierarchical clustering show the relationship between key bacterial genera and SCFA production for VH and ABX (Fig. 3c, d). Specifically, the production of butyrate, propionate, 3-methyl-valeate and are associated with the abundance of Erysipelatoclostridium (q = 6.2e-3, q = 0.045, and 3.3e-3 respectively), and the increment of 2-methyl-butyrate levels are associated to Alistipes (q = 0.039) (Fig. 3c). This analysis highlights the intricate relationships between SCFA profiles and bacterial genera, suggesting that ABX treatment following TBI does not significantly affect these dynamics (Fig. 3d).

### Deletion of the gut microbiota alleviates the neuroprotective effect in mice with TBI.

**Figure 4:**
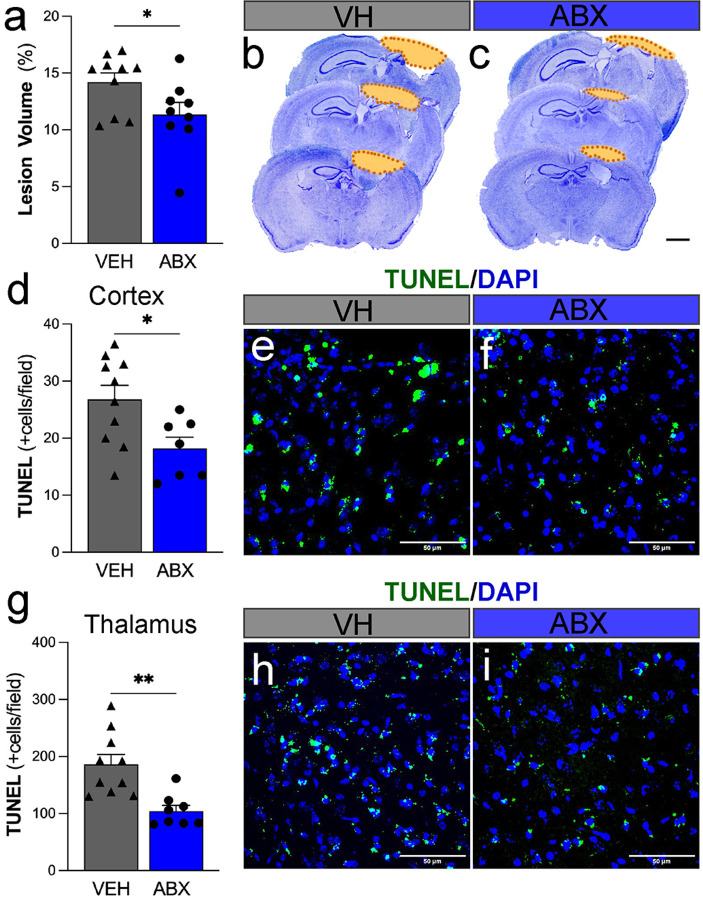
Antibiotic (ABX) Treatment reduces Lesion Volume and Cell Death Post-Traumatic Brain Injury (TBI). **(a)** Cortical lesion volume as a percentage of total brain volume displaced a 25% decrease in ABX-treated mice compared to in VH-treated (VH) mice 5 days post-second TBI. Representative Nissl-stained brain sections from VH-treated **(b)** and ABX-treated **(c)** brains. The dotted lines delineate the lesion area, with the damaged region noticeably smaller in the ABX-treated group. **(d)** Quantification of TUNEL-positive apoptotic cells per field in the cortex **(d, e, f)** and thalamus **(g, h, i)** regions at 5 dpi. Representative fluorescent microscopy images of cortical regions stained with TUNEL (green) and DAPI (blue). Mean ± SEM. n=9/group. * (p<0.05), ** (p<0.01). Scale bars in (b, c) = 1 mm, and in (e, f, h, i) = 50 μm.

To eliminate the influence of gut microbiota differences on experimental results, animals were treated with ABX for 2 days before the second TBI. Subsequently, the effects of ABX treatment on lesion volume and apoptotic cell death after TBI were examined using TUNEL staining and histological analysis. The cortical lesion size was significantly 25% smaller in the ABX-treated group compared to the VH group (Fig. 4a) at 5 dpi. Representative histological images of the cresyl-violet brain sections showed the reduced lesion area in the ABX group (Fig. 4a, c). TUNEL staining, used to detect apoptotic cells in the injured brain. In the cortex, the ABX group had fewer TUNEL-positive cells (18.2 ± 1.9) than the VH group (26.8 ± 2.4), indicating reduced cellular loss (Fig. 4d). Similar trends were observed in the thalamus (Fig. 4g), where the ABX group exhibited significantly fewer TUNEL-positive cells (104.8 ± 9.8), than the VH group (186.2 ± 17.3). The representative TUNEL staining images (Fig. 4e–f, h–i) further demonstrate these findings, showing reduced apoptotic cells in the ABX group. These results suggests that ABX therapy might offer neuroprotective benefits in mitigating brain damage and cell death post-TBI.

### Microbiome depletion alters microglia/macrophage activation and morphology after TBI.

**Figure 5:**
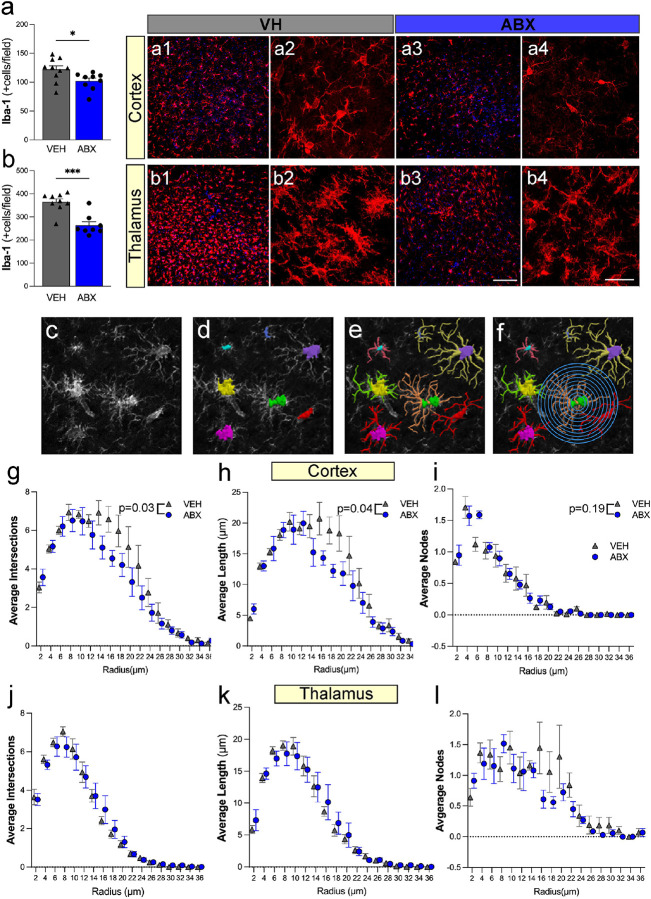
ABX reduce the microglia/macrophage density and morphology in the cortex and thalamus following TBI. Bar graphs represent the quantification of Iba-1 positive cells in the cortex **(a)** and thalamus **(b)**, respectively, comparing VH and ABX-treated groups post-TBI. Representative images of Iba-1 staining in the cortex **(a1-a4)** and thalamus **(b1-b4)**in VH and ABX-treated groups**. (c)** shows the raw image of Iba-1 positive stained microglia/macrophages (Iba-1), **(d)** displays the same image with microglia somas highlighted in different colors for individual analysis, (e) illustrates the traced microglial/macrophages dendritic processes, and **(f)**presents a Sholl analysis with concentric circles to analyze process complexity. **(g-l)** Graphs displaying the results of Sholl analysis in the cortex and thalamus for VH and ABX-treated groups, with **(g, j)** showing the average number of intersections, **(h, k)** the average process length, and **(i, l)** the average number of nodes as a function of distance from the cell body. Mean ± SEM. n=9/group. * (p<0.05), *** (p<0.001). Scale bar: 50 μm **(a1, b1, a3, b3)** and 20 μm **(a2, b2, a4, b4,** c- f).

Microglial density and morphology were analyzed in the cortex and thalamus following the second TBI in VH and ABX-treated groups. Quantification of Iba-1-positive cells showed a significant decrease in both the cortex (101.9 ± 4.9, p = 0.03) (Fig. 5a) and thalamus (263.6 ± 20.7, p < 0.001) (Fig. 5b) of ABX mice as compared to the VH (121.9 ± 6.4 and 364.7 ± 13.6, respectively). Representative Iba-1 staining images in the cortex and thalamus are shown in panels (Fig. 5a1–a4) and (Fig. 5b1–b4), respectively, demonstrating the morphological differences between VH and ABX-treated groups. To further analyze microglia processes and morphology following TBI, a Sholl analysis of microglial structural characteristics was assessed by plotting the radial distance of corresponding dendrite intersections, lengths, and nodes, from the soma. First, a representative Iba-1 image was acquired through confocal imaging (Fig. 5c). Individual microglia somas were established (Fig. 5d) before manually mapping dendrite branching (Fig. 5e). Lastly, the Sholl analysis was conducted for each microglia by adding concentric circles 2um apart, starting at each microglia soma (Fig. 5f) to evaluate process complexity. The specific measurements of the number of dendritic intersections, dendrite branching length, nodule processes, and microglia surface area were measured and plotted using Neurolucida software. The Sholl analysis results (Fig. 5g–l) in the cortex and thalamus indicate significant differences in microglial process intersections, average process length, and average nodes. There was a significant decrease in the overall average number of intersections (24.6 ± 9.5, p = 0.03) and average dendrite length (70.7 ± 29.4, p = 0.04) in the cortical region of mice that received ABX compared to the VH mice (Fig. 5g, h). No other significant morphological changes were present in the average number of nodes and average surface area in the somatosensory cortex (Fig. 5i), and no significant changes overall in the thalamus (Fig. 5j, k, l). These results suggest that ABX treatment after TBI reduces microglial/macrophage density and alters morphology, with fewer processes and reduced complexity, indicating a potentially reduced inflammatory response compared to the VH-treated group.

### ABX treatment reduces microglia/macrophage activation and astrogliosis but does not affect neutrophil infiltration following TBI.

**Figure 6:**
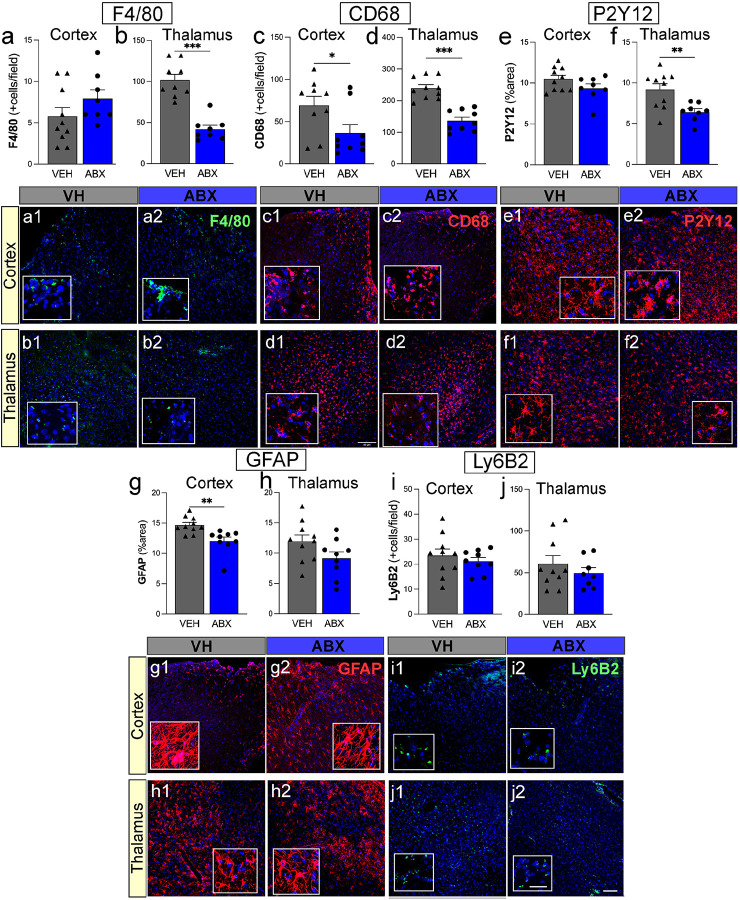
ABX treatment decrease microglia/macrophage activation and astrogliosis but does not affect neutrophil infiltration following TBI. **(a, b)** F4/80+ macrophage/microglial cells, **(c, d)** CD68+ microglial/macrophage activation marker, and **(e, f)** P2Y12+ resident microglial cells. **(a1- f2)**Fluorescent immunohistochemistry images illustrate the expression of F4/80, CD68, and P2Y12, in the cortex **(a, c, e)** and corresponding markers in the thalamus **(b, d, f)** for VH and ABX-treated groups, with insets showing higher magnification. Nuclei are stained with DAPI (blue), and the inflammatory markers are shown in red or green. **(g- j)** Bar graphs quantify the expression of GFAP, a marker of astrocyte activation, and Ly6B2, a neutrophil marker, in the cortex **(g, i)** and thalamus **(h, j). (g1-j2)** Corresponding fluorescent immunohistochemistry images for GFAP **(g)** and Ly6B2 **(i)** in the cortex and GFAP **(h)** and Ly6B2 **(j)** in the thalamus, with insets for detailed view. GFAP is presented in red, Ly6B2 in green, and DAPI-stained nuclei in blue. Insets show higher magnification, with nuclei stained with DAPI (blue) and the inflammatory markers in red. Scale bars: main images = 50 μm, insets = 20 μm. * Mean ± SEM. n=9/group. *(p<0.05), ** (p<0.01), and *** (p<0.001).

The antibody F4/80, a well-characterized membrane protein highly expressed in murine macrophages, displayed a significant decrease in only the thalamus of ABX (41.9 ± 4.8, p < 0.001) compared to VH (101.7 ± 7.0) mice. There were no significant changes in the F4/80 positive macrophage/microglial cells observed in the somatosensory cortex (Fig. 6a, b). We found that there was a significant decrease in CD68 positive microglial/macrophage activation in both the somatosensory cortex (36.5 ± 9.7, p = 0.04) and thalamus (136.4 ± 11.2, p < 0.001) in ABX treated mice compared to the VH group (69.2 ± 10.7, 239.6 ± 10.7, respectively) (Fig. 6c, d). There was a significant decrease of P2Y12 positive resident microglial cells in the thalamus region of the brain in ABX-treated mice (6.4 ± 0.4, p = 0.006) versus VH-treated mice (9.1 ± 0.7), however, there was no significant change in the percent area of stained cells in the cortical region (Fig. 6e, f). The data show a significant reduction in GFAP expression in the ABX-treated group (12.0 ± 0.6, p = 0.003) compared to VH mice in the cortex (14.6 ± 0.4), suggesting decreased astrogliosis. The percentage area of activated astrocytes was not significantly changed in the thalamus region between ABX and VH mice (Fig. 6g, h). Neutrophil Infiltration was measured by the number of Ly6B2 positive cells per field in the cortex and thalamus, with no change in infiltration in either region between ABX or VH-grouped mice at 5 days post-second TBI (Fig. 6i, j). Fluorescent immunohistochemistry images show the expression of F4/80 (Figure a1, a2, b1, b2), CD68 (Fig. 6c1, c2, d1, b2), P2Y12 (Fig. 6e1, e2, f1, f2), GFAP (Fig. 6g1, g2, h1, h2), and Ly6B2 (Fig. 6i1, i2, j1, j2) in the cortex and the thalamus. These results suggest that while ABX treatment after TBI decreases microglia/macrophage activation and astrogliosis, it does not appear to affect neutrophil infiltration.

### Impact of antibiotic treatment on gut histology following traumatic brain injury.

**Figure 7:**
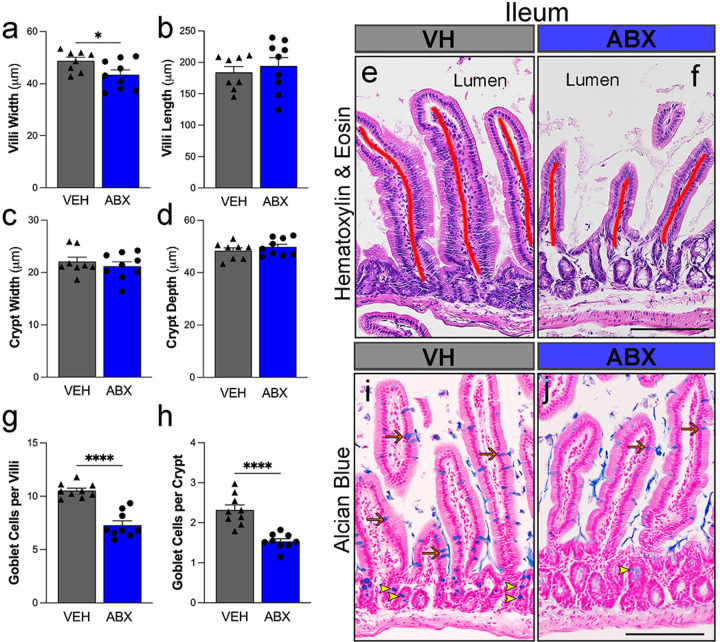
ABX significantly alters gut morphology and decrease goblet cells following TBI. (a) **(a)** Villi width and **(b)** length were quantified, demonstrating a reduction in villi width in antibiotic treatment (ABX) compared to vehicle (VH) mice. Crypt width **(c)** depth **(d)** measurements show no significant differences between treatments. Representative hematoxylin and eosin-stained sections of the damage small intestines after TBI from VH **(e)** and ABX **(f)**treated mice, illustrating the overall architecture and cell morphology. Lines in the image in red indicated the length of the villi. The ABX-treated group shows a significant decrease in goblet cell count in the villi **(g)**and crypts **(h)** of the ileum. Alcian blue (blue) and nuclear fast red (pink) staining of the small intestine sections from VH **(i)** and ABX (j)treated mice, highlighting the mucin-producing goblet cells per crypt (yellow head arrow) and goblet per villi (orange arrow). Scale bars: (e, f, I, j) = 100 μm. Mean ± SEM. n=9/group. *(p<0.05), and *** (p<0.001).

We sought to determine the effects of ABX treatment on overall small intestinal microarchitecture (villi length/width and crypt depth/width) that impacts nutrient absorption. Small intestine samples were stained with hematoxylin and eosin to label intestinal villi and crypt structure and with Alcian blue to measure goblet cell and mucin production. ABX treatment induced a significantly shorter villi width (Fig. 7a, 43.3 ± 1.8, p = 0.04) versus VH (48.7 ± 1.3). No significant differences in crypt and villi length (Fig. 7c, d) were found between ABX and VH groups. Next, we examined the impact of ABX treatment on goblet cells and mucin production. These cells are key components of the mucosal barrier and play important roles in host defense against intestinal pathogens. Compared to controls, ABX-treated mice had a significantly fewer goblet cells per field (Fig. 7g, 7h), in both the villi (7.3 ± 0.4, p < 0.001) and crypt (1.5 ± 0.06, p < 0.001) regions of the small intestines versus VH-treated (10.5 ± 0.2 and 2.3 ± 0.1, respectively). These results suggest that ABX treatment after TBI induces significant changes in gut histology, notably broader villi and a reduction in goblet cells.

**(a)** Villi width and **(b)** length were quantified, demonstrating a reduction in villi width in antibiotic treatment (ABX) compared to vehicle (VH) mice. Crypt width **(c)** depth **(d)** measurements show no significant differences between treatments. Representative hematoxylin and eosin-stained sections of the damage small intestines after TBI from VH **(e)** and ABX **(f)** treated mice, illustrating the overall architecture and cell morphology. Lines in the image in red indicated the length of the villi. The ABX-treated group shows a significant decrease in goblet cell count in the villi **(g)** and crypts **(h)** of the ileum. Alcian blue (blue) and nuclear fast red (pink) staining of the small intestine sections from VH (i) and ABX (j) treated mice, highlighting the mucin-producing goblet cells per crypt (yellow head arrow) and goblet per villi (orange arrow). Scale bars: (e, f, I, j) = 100 μm. Mean ± SEM. n = 9/group. *(p < 0.05), and *** (p < 0.001).

## Discussion

In our research, we observed that a broad-spectrum ABX treatment significantly altered the composition of the fecal microbiota following TBI in mice (Figs. 1, 2). Our results revealed that ABX treatment resulted in significant shifts in the relative abundance of various bacterial taxa at both the phylum and genus levels, underscoring the profound impact of ABX therapy on gut microbial communities.

We administered a combination of antibiotics (ampicillin, gentamicin, metronidazole, and vancomycin) to deplete the gut microbiota. Ampicilin-induced changes in the microbiota, decreasing the bacterial diversity, the expression of MHC class I and II genes, and elevated mast cell protease expression in the intestine [36]. Mice treated with gentamicin shifted intestinal community and impacts in the gut metabolome decreasing SCFA [37]. Metronidazole is an antimicrobial that selectively targets anaerobic bacteria [38], and it has been shown to affect the gut microbiota by slightly decreasing the *Firmicutes to Bacteroidetes* ratio, primarily due to an increase in *Bacteroidetes* [39]. Finally, vancomycin, a narrow-spectrum ABX effective against Gram-positive bacteria like *Staphylococcus* and *Clostridium* and has been shown to diminish microbiome diversity and change its composition, reducing the production of SCFAs [14].

We found a clear split in bacterial diversity one day after ABX treatment, with the VH group exhibiting more uniformity while the ABX-treated group displayed greater disparity by 5 dpi. These shifts after ABX treatment indicate a transition from a balanced gut microbiome toward a more dysbiotic state. We noted that alpha and beta diversity differed only following ABX treatment, maintaining uniformity throughout the study. However, pronounced beta diversity changes following ABX treatment has been previously observed by others in studies on microbiome disruption in adolescent and adult rats following TBI [40]. In clinical studies, factors such as ABX exposure and the occurrence of infection were linked to larger disparities in the rectal and oral microbiomes between TBI patients and healthy controls [41].

Our findings revealed several significant shifts in the fecal microbiome in the acute and chronic phases after the first TBI. Notably, we observed an increase in the abundance of genus *Lachnospiraceae* UCG-006 during both phases compared to baseline levels. The genus *Lachnospiraceae* UCG-006 is known to produce SCFAs, such as butyrate, which have anti-inflammatory properties [42]. This aligns with previous studies that suggest a positive correlation between Lachnospiraceae UCG-006 and antioxidant enzyme activity [43]. A higher relative abundance of the genus *Lachnospiraceae UCG-006* is associated with positive health outcomes after infection with *S. Typhimurium* [32]. Thus, the increase in this genus could suggest its contribution to repair processes after TBI, potentially lessening in ammation and enhancing recovery. This increase was accompanied by a significant reduction in *Bifidobacterium and Lactobacillus*, corroborated by our previous research [44]. *Bifidobacteria* were significantly negatively correlated with the expression of cortical genes related to inflammatory responses. [45].

In the acute stage of the first TBI, we found a marked decrease in beneficial bacteria like *Bifidobacterium* and *Lactobacillus* (Fig. 2i), as we have previously published [44]. Similarly, we observed a reduction in Enterorhabdus during the chronic phase (Fig. 2j). Interestingly, the *Enterorhabdus genus* includes pathogenic bacteria that were first isolated from a mouse model of intestinal inflammation [46]. In an animal model of dietary-induced nonalcoholic fatty liver disease (NAFLD), the probiotic *L. plantarum NA136 treatment* relieved insulin resistance and significantly increased relative proportions of *Enterorhabdus* [47]. Studies using mouse models have demonstrated that specific probiotic bacteria, like Lactobacillus, can enhance vagus nerve activity, modify brain neurotransmitter levels of GABA, glutamate, and serotonin, and reduce anxiety-related behaviors [48]. Similarly, since both *Bifidobacterium* and *Lactobacillus* can produce GABA following TBI in mice [45], it is plausible to consider that they may also act as neuroprotective probiotics as we have demonstrated in our previous work [49]. Another study found that *Lactobacillus* may play a neuroprotective role by reshaping the intestinal microbiota of TBI mice [50]. These changes suggest that TBI can disrupt the balance of beneficial and harmful bacteria, potentially impacting brain recovery. In Group I of mice, which were not treated with ABX 38 days post-first TBI, there was an observed increase in *Ruminococcus*. This genus is also recognized for producing SCFAs, especially butyrate, suggesting a potential role in reducing inflammation and aiding brain recovery [51]. However, our metabolite data did not show a correlation with any of the increased SCFAs analyzed.

After the second TBI, the ABX-treated group experienced acute dysbiosis, with a decrease in *Lolidextribacter, Akkermansia, Bacteroides, Lachnoclostridium*, and *Parasutterella,* alongside a slight increase in *Duboisella*. A decline in gut barrier-protective bacteria like Akkermansia could lead to a more permeable gut lining [52], allowing inflammatory mediators to enter the bloodstream and potentially reach the brain, further exacerbating neuroinflammation. Treatment with A. *muciniphila* was shown to improve intestinal injury, neurological dysfunction, and neuroinflammation in the cerebral cortex of mice with TBI [53]. Previous clinical studies have shown that individuals with lower bacterial diversity before receiving ABX may be more vulnerable to opportunistic or pathogenic species, such as *Lachnoclostridium* [54]. An increase in *Duboisella*, given the overall context of dysbiosis, could indicate a shift in microbial balance, indicating ongoing disruption and a potential risk of further inflammatory conditions [55]. These findings show that depleting the pathogenic microbiome could be a therapeutic target to decrease inflammation and help to the brain recovery.

ABX decrease levels of acetate, n-butyrate, and propionate, which are products of microbial fermentation, and reduce adenine, cytosine, guanine, and uracil due to an overall reduction in bacterial load [28]. In our study, we did not find no changes in neuroprotective SCFA levels after ABX treatment, though neuroinflammation and injured brain regions were decreased, likely through mechanisms not directly tied to SCFAs. *Erysipelatoclostridium* are inflammation-associated microbes, and they contributed significantly to metabolites synthesis in the gut [56]. Butyrate, considered the most significant SCFA, is produced by a broad phylogenetic array of bacteria. This includes many types of *Firmicutes,* such as *Ruminococcus, Clostridial Clusters* IV and *XIVa, Eubacterium, Anaerostipes, Coprococcus, Faecalibacterium,* and *Roseburia*, as well as certain species of *Bacteroides* and *Bifidobacterium* [57]. Our analysis also shown butyrate and propionate as key metabolites associated with *Erysipelatoclostridium* as an essential gut bacterial genus. Furthermore, in TBI mice treated with VH, serum butyrate and propionate levels were positively correlated with the abundance of *Erysipelatoclostridium* and negatively correlated with levels of 2-methyl-butyrate. This indicates that five days after TBI, the microbiota capable of producing SCFAs is replenished in animals not subjected to antibiotic treatment, promoting recolonization with beneficial bacteria. This restoration does not happen in the group treated with ABX.

We utilized short read 16S rRNA amplicon profiling to analyze microbial populations, which allowed us to identify and assess the relative abundance of individual bacterial taxa. While we employed best practice microbiome analysis tools for short read 16S analysis [58] analyzing changes in microbiome data due to limited resolution of short regions of 16S limit our analysis down to genus levels [59]. While this provides a high-level view of the bacterial communities inhabiting the mouse gut, it does not allow us to interrogate specific species and/or strains directly responsible for metabolite and SCFA production interfacing with the gut-brain axis Also, given the compositional nature of microbiome analysis, relative abundance analysis can introduce bias and measurement error [60].

The gut microbiota has been shown to influence BBB permeability [61]. The host-microbiota influences brain levels of numerous neurochemical mediators crucial for neuronal function [62]. Gut microbiota regulates the innate immune functions of microglia, which prepares the brain to respond rapidly to pathogens or threats. After administering ABX, it was observed that the intestinal microbiota influenced the neuroinflammatory response and brain injury following neonatal hypoxia-ischemia in mice [63]. Another study showed that administering ABX prior to TBI in mice helped lessen early neuroinflammatory responses, yet it did not significantly affect brain histopathology [64]. Furthermore, the lack of a complex host microbiota leads to deficiencies in microglia maturation, differentiation, and function. Consequently, mice treated with germ-free (GF) and ABX conditions showed impairments in microglial functionality [12]. Our neuropathological analysis revealed that microglia/macrophage cells and apoptotic cells were significantly lower in ABX-treated mice, indicating potential neuroprotective benefits, align with existing studies showing that the depletion of gut bacteria can attenuate the neuroinflammatory response [65]. Other studies have shown that although ABX may contribute to a reduction in lesion volume following TBI, it adversely reduces monocyte infiltration, worsens neuronal loss, and increases microglial pro-inflammatory markers following a single TBI [66]. Despite the neuroprotective effects of ABX, our results did not show significantly alter neutrophil infiltration in the cortex and thalamus, indicating that certain aspects of the inflammatory response remain unaffected.

ABX treatment had a notable impact on gut pathology, leading to significant changes in the small intestine’s structure. Clinical data on mucosal barrier function following TBI are sparse and limited to ICU patients [17]. Goblet cells, specialized epithelial cells in the gastrointestinal tract responsible for producing and secreting mucin, play a critical role in maintaining the mucus barrier. A reduction in goblet cell count can weaken this barrier, which could result in immune dysregulation and chronic inflammation. Our findings showed a significant decrease in goblet cell count indicating a disruption in the gut’s protective barrier. This shift suggests that ABX treatment can impact gut homeostasis, possibly disrupting mucin production and other protective mucus components.

## Conclusions

Collectively, this study indicates that ABX treatment following TBI has complex effects, including shifts in gut microbiome diversity and composition, reduced neuroinflammation, and changes in gut pathology. Further research is needed to understand the long-term effects of ABX treatment on brain-gut interactions and post-TBI recovery to help optimize therapeutic strategies for TBI patients, aiming to reduce neuroinflammation while preserving gut health.

## Data Availability

The datasets generated in this study are stored in the SRA database from NIH with the BioProject number: PRJNA1104663. All other data are available from the authors upon reasonable request.
